# Biaryl scaffold-focused virtual screening for anti-aggregatory and neuroprotective effects in Alzheimer’s disease

**DOI:** 10.1186/s12868-018-0472-6

**Published:** 2018-11-13

**Authors:** Sidra Khalid, Muhammad Ammar Zahid, Hussain Ali, Yeong S. Kim, Salman Khan

**Affiliations:** 1 Department of Pharmacy, Faculty of Biological Sciences, Quaid-i-Azam University, Islamabad, 45320 Pakistan; 2 Natural Products Research Institute, College of Pharmacy, Seoul National University, Seoul, South Korea; 3 Department of Biotechnology, Faculty of Biological Sciences, Quaid-i-Azam University, Islamabad, Pakistan

**Keywords:** Computational analysis, Alzheimer, Biaryl scaffold, Neuroprotection

## Abstract

**Background:**

Alzheimer’s disease (AD) is a primary cause of dementia in ageing population affecting more than 35 million people around the globe. It is a chronic neurodegenerative disease caused by defected folding and aggregation of amyloid beta (Aβ) protein. Aβ is formed by the cleavage of membrane embedded amyloid precursor protein (APP) by using enzyme ‘transmembrane aspartyl protease, β-secretase’. Inhibition of β-secretase is a viable strategy to prevent neurotoxicity in AD. Another strategy in the treatment of AD is inhibition of acetylcholinesterase. This inhibition reduces the degradation of acetylcholine and temporarily restores the cholinergic function of neurons and improves cognitive function. Monoamine oxidase and higher glutamate levels are also found to be linked with Aβ peptide related oxidative stress. Oxidative stress leads to reduced activity of glutamate synthase resulting in significantly higher level of glutamate in brain. The aim of this study is to perform in silico screening of a virtual library of biaryl scaffold containing compounds potentially used for the treatment of AD. Screening was done against the primary targets of AD therapeutics, acetylcholinesterase, β-secretase (BACE1), Monoamine oxidases (MAO) and *N*-Methyl-D-aspartate (NMDA) receptor. Compounds were screened for their inhibitory potential by employing molecular docking approach using AutoDock vina. Binding energy scores were embodied in the heatmap to display varies strengths of interactions of the ligands targeting AD.

**Results:**

Several ligands showed notable interaction with at least two targets, but the strong interaction with all the targets is shown by very few ligands. The pharmacokinetics of the interacting ligands was also predicted. The interacting ligands have good drug-likeness and brain availability essential for drugs with intracranial targets.

**Conclusion:**

These results suggest that biaryl scaffold may be pliable to drug development for neuroprotection in AD and that the synthesis of further analogues to optimize these properties should be considered.

**Electronic supplementary material:**

The online version of this article (10.1186/s12868-018-0472-6) contains supplementary material, which is available to authorized users.

## Background

**Fig. 1:**
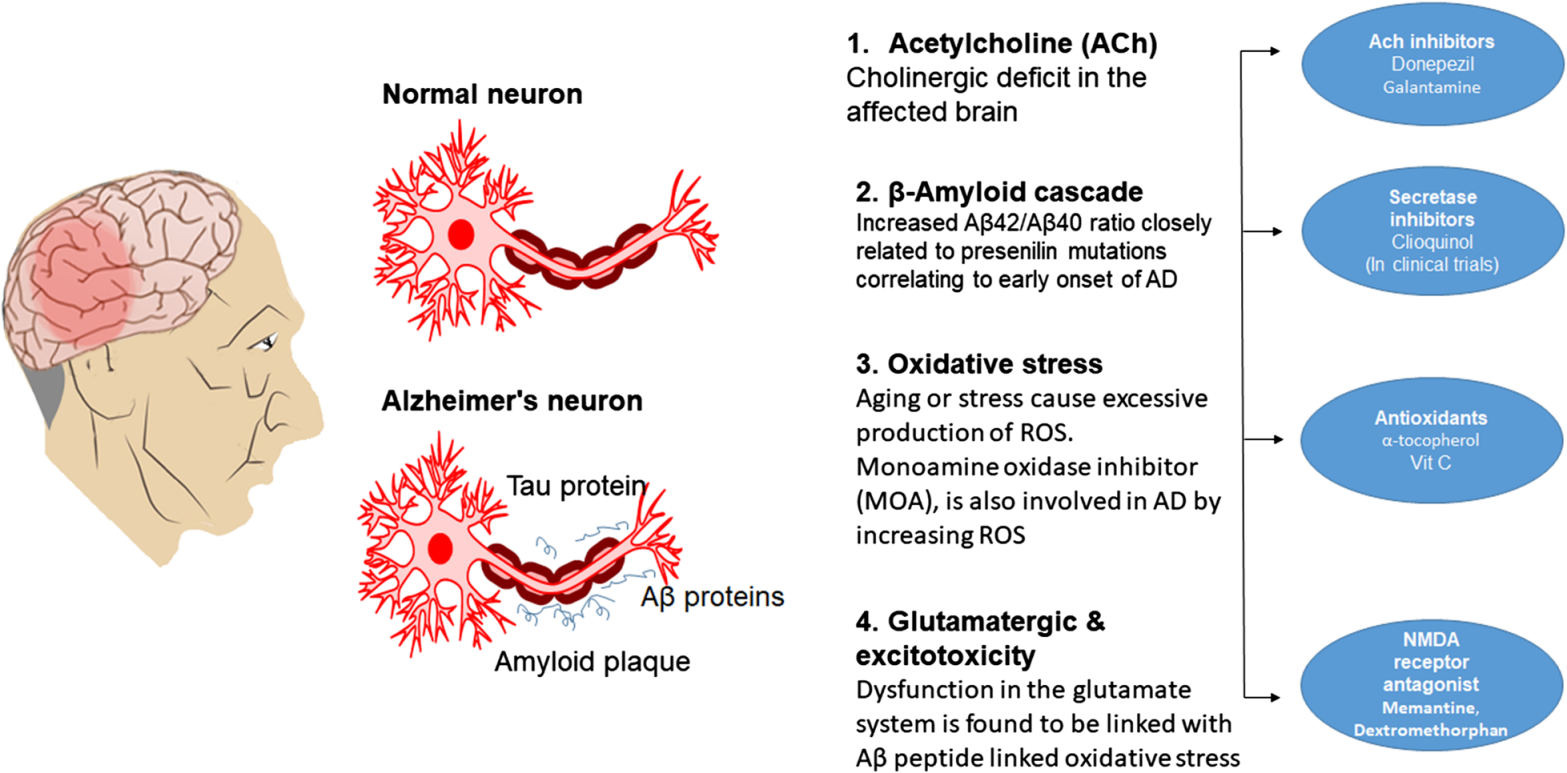
Pathogenesis of Alzheimer disease (AD) and therapeutic intervention are shown. (1) Acetylcholine (ACh) inhibitor activation leads towards ACH deficit in effected brain and drugs that inhibits acetylcholinesterase (Donepezil). (2) Aβ generation and aggregation and its sites for therapeutic intervention. All the drugs currently used in this regard is in clinical trial phase. (3) Oxidative stress; ROS can aggravate and trigger AD, Antioxidant maybe helpful. (4) Glutamatergic dysfunction and excitotoxicity play role in pathogenesis of AD. NMDA receptor antagonist (Memantine) are the treatment option

Alzheimer disease (AD) is the primary cause of dementia worldwide. Currently, more than 35 million people are suffering from this disease around the globe. By the year 2050, the diseases burden is excepted to raise four times i.e. almost 1 out of 85 persons will be suffering from AD [1]. The major pathological hallmarks of AD include widespread neuronal and synaptic loss, excessive presence of astrocytes, and aggregation of multiple proteinaceous deposits for instance β-amyloid plaques and neurofibrillary tangles (NFT) [2]. The number of hypotheses are proposed along the years to describe the root cause of AD such as the production of β-amyloid, cholinergic hypothesis, excitotoxicity and oxidative stress hypothesis [3] as summarized in Fig. 1. Senile plaques are the main and distinguished neurological feature of the AD directly related to its onset and progression [4]. The production of amyloid beta (Aβ) takes place by the proteolytic cleavage of beta-secretase protein on amyloid precursor protein (APP) whereas in AD, a pathogenic mutations affects the protease cleavage sites in APP and aid its cleavage [5]. The Aβ are mainly divided into two isoforms, based upon the length of amino acids, Aβ of 40 amino acid residues (Aβ40) and Aβ of 42 amino acid residues (Aβ42) are the two isoforms. Although Aβ42 differ in only 2 amino acids, it is much more neurotoxic and aggregates faster as compared to Aβ40. In the cerebrospinal fluid (CSF) presence of Aβ42 is a well-known biomarker of AD, and is used both in AD research and increasingly in clinical practice [6]. The increased level of Aβ42 as compare to Aβ40 has been generally considered to play a critical role. The increased Aβ42/Aβ40 ratio is closely related to presenilin mutations correlating to early onset of AD [7]. Although Aβ40 is several-fold more abundant in the brain Aβ42 is the major and sometimes exclusive component in amyloid plaques, due to its more aggregation prone nature [7].

AD is also characterized by the cholinergic deficit in the affected brain. The acetylcholine-releasing neurons especially there cell bodies which lied in basal forebrain degrades selectively in AD affecting cognitive functions and memory as these neurons are vital in the normal functioning of cerebral cortex and related structures. In AD there is a modification and alteration in polymorphism of acetylcholinesterase (AChE) in brain [8]. An increased amount of AChE levels around the Aβ plaques and NFT is commonly reported feature of AD. The current therapy of AD is mainly based on the use of AChE (AChE-I) inhibitors. The effect of these AChE-I is modest and transient due to up-regulation of AChE activity following chronic AChE-I therapy [9].

Another mechanism by which AD can develop is by excessive presence of the reactive oxygen species (ROS) in the mitochondria. This rise in ROS is due to aging or stress and if the antioxidant system of the body fails to cope with this condition AD may develop. The role of oxidative stress in AD is evident from the fact that brain of these patients shows a substantial oxidative damage [10]. Monoamine oxidase (MOA), is also involved in AD by increasing ROS in brain. MAO and its isoenzymes i.e. MAO-A and MAO-B are liable for the catalysis of biogenic amines, like serotonin, dopamine and norepinephrine so its inhibition results in an augmented level of neurotransmitters in the CNS [11].

Similarly, the key excitatory neurotransmitter glutamate which is involved in synaptic plasticity and learning, is also linked to AD. Dysfunction in the glutamate system is found to be linked with Aβ peptide linked oxidative stress. Oxidative stress leads to reduced activity of glutamate synthase resulting in significantly higher level of glutamate in brain [12]. The over-activation of *N*-methyl-D-aspartate receptor (NMDA) receptor due to excessive glutamate leads to the continuous calcium ions (Ca^2^+) influx into the nerve cells, generating a slow excitotoxicity at post synaptic level ultimately leading to a gradual neurodegenerative effect in AD patients [13]. Thus, NMDA receptor antagonists could be advantageous therapeutically in the management of AD.

Inhibition of these targets individually with currently approved or developing drugs has been proved relatively unsuccessful at reversing the progression of AD. A likely solution lies in a multi-pharmacological approach to altered activities of numerous of these targets at the same time, particularly those associated with the progression of the disease. Such multiple target drugs developed for AD have targeted two or more of known targets (cholinesterases, BACE1, MAO, NMDA) or have disease progression retarding properties, such as metal chelation, reduce oxidative stress or have anti-inflammatory potential, or can prevent Aβ or tau aggregation [14]. Ligands for drug targets combinations should be assessed against disease progression to define best possible combinations.

Molecular docking; a computational technique, is used for the estimation of the binding affinity between two molecules like the protein–protein and ligands-protein [15]. Virtual screening or computer-aided drug design (CADD) combined with wet lab techniques contributes towards the development of new drug molecules [16]. CADD is particularly useful especially in three major areas: (1) selection of most suitable compounds from large libraries of possibly actives compounds (2) Addition of an appropriate functional groups in the lead compounds for making it more suitable for new drug development (3) By adding pharmacophore features, designing the new molecules from a target structure. In light of current study the interactions of selected compounds with the various targets of AD was determined, using docking and in silico absorption, distribution metabolism and excretion (ADME) pharmacokinetics studies [15].

## Methods

### Preparation of protein targets

The target proteins i.e. AChE (4EY7) [17], BACE-1 (2HM1) [18], MAO-A (2Z5X) [19] and NMDA (1PBQ) [20] were selected. These X-ray crystallographic structures were downloaded from protein data bank (PDB). Preparation of all the protein structure was done in Chimera by using ‘Dockprep’ workflow [21]. The preparation includes addition of hydrogen to the protein, assignment of bond orders, and unnecessary associated molecules deletion. Addition of side chains was done, partial charges were assigned, disulphide bonds were made and missing atoms were added. Optimized Potentials for Liquid Simulations (OPLS_2005) force field was used for energy minimization. The active sites of the proteins were determined by the co-crystallized ligands.

### Ligand dataset preparation

The zinc database (zinc15.docking.com) was searched for the biaryl scaffold. The hits were filtered and only those compounds were selected for further processing which was ever tested in vivo (neither in animal model nor in man) and available for free sale. The ids of compounds along with their binding affinities are also present in the attached file (Additional file 1). Resulting molecules were downloaded in mol2 format. Ligand’s pre-processing was done, using Ligprep, the formation of tautomers and ionization states (pH 7.0 ± 2.0) using Epik [22]. An addition of hydrogen atoms was also done, neutralization of charged groups and geometry of the ligands were also optimized.

### Virtual screening: binding mode analysis

Computational analysis was performed by firstly downloading mol2 structures of the ligands from ZINC database and then converted to PDBQT formats after assigning Gastegier charges and merging non-polar hydrogens by using AutoDock Tools 1.5.4. PyRX software was used for virtual screening. Both Autodock and AutoDock Vina are included in the PyRX [24]. The binding site for docking analysis was determined by the position of the co-crystallized ligand. The grid box was centered on the experimentally docked ligands with the dimension given in Table 1. Docking was performed using AutoDock Vina (version 1.1.2) by considering all the bonds in the ligands as rotatable and the proteins as the rigid structures. Rest of the parameters were kept as default and docking scores were calculated by the default scoring function [23]. The best binding modes of the ligands were exported as mol2 files and the interaction of the best binding modes with the protein target were investigated by using discovery studio visualizer. Ligands with best binding scores were redocked using the glide/SP docking algorithm in Maestro https://pubs.acs.org/doi/10.1021/jm0306430. The binding poses generated by glide were matched with the best binding poses from AutoDock Vina using an RMSD cutoff of Å. All RMSD values were calculated using the python script “rmsd.py”. Data warrior was used for data handling and visualization [25]. To find out compounds with multi-target binding efficiency, the heatmap was generated using chemmines numerical clustering tool based on binding scores [26].

**Table 1:** Grid box centre and dimension for docking of the ligands against target protein

Protein	Grid box centre	Grid box dimensions
4EY7	X: − 2.91	X:59.75
	Y: − 40.11	Y:61.25
	Z: 30.86	Z:72.51
2HM1	X: 16.08	X:57.15
	Y: − 0.07	Y:68.97
	Z: 10.02	Z:48.99
1PBQ	X: 2.535	X:55.36
	Y: 39.35	Y:49.88
	Z: − 17.65	Z:48.12
2Z5X	X: 34.6965	X:88.4415
	Y: 28.131	Y:75.9847
	Z: − 20.0943	Z:62.8238

Where: acetylcholinesterase (4EY7), beta-secretase cleavage enzyme (2HM1), monoamine oxidase (2Z5X) and *N*-methyl-D-aspartate receptor receptor (1PBQ)

### Pharmacokinetic parameters

The calculation of the physiochemical properties of the drugs is done by SwissADME. Physiochemical properties like, octanol/water partition coefficient (XPlogPo/w), compound’s molecular weight (MW), the number of hydrogen bond acceptors (accptHB), hydrogen bond donors (donorHB), and percentage human oral absorption, blood brain penetration was predicted. Violations of Lipinski’s rule of five by any drug was also analyzed. Based on these molecular descriptors, the intestinal absorption and blood–brain barrier penetration were represented by using a BOILED-egg model [27].

## Result

**Fig. 2:**
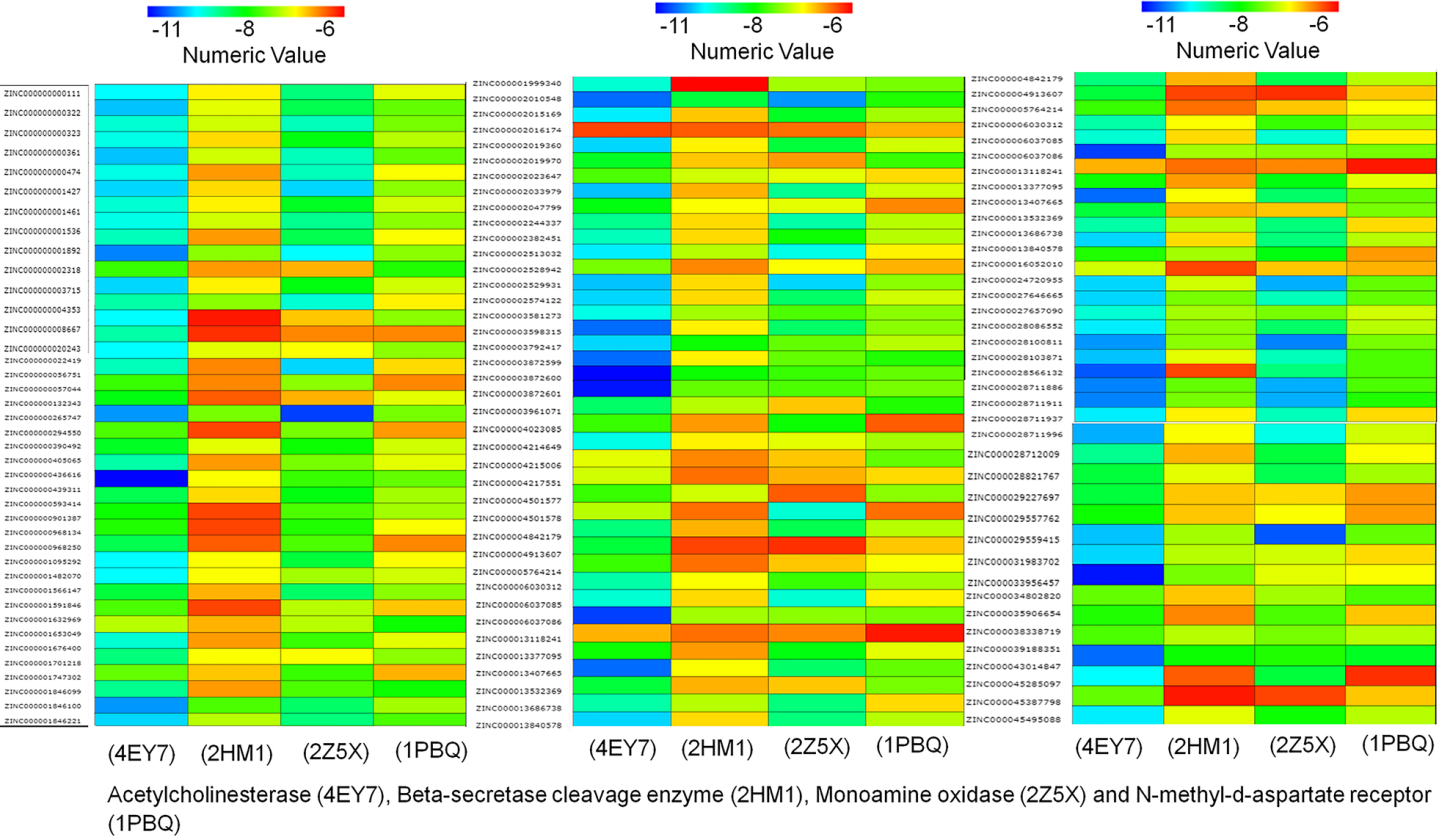
The heatmaps based on binding scores compounds with multitarget binding efficiency. The heatmap was generated using chemmines numerical clustering tool based on binding scores. A heat map analysis of binding constants of 107 in vivo tested compounds screened against acetylcholinesterase (4EY7), beta-secretase cleavage enzyme (2HM1), monoamine oxidase (2Z5X), *N*-methyl-D-aspartate receptor receptor (1PBQ) by AutoDock tool. In the gradient ruler, blue colour indicated strong binding (Docking score − 11), while red colour indicate weak binding (Docking score − 6)

The key focus of the current study is to identify new compounds containing biaryl scaffold for the treatment of AD. Around, 107 compounds were screened using in silico molecular docking technique by AutoDock Vina. Out of the screened compounds, ZINC000003872600, ZINC000002010548, ZINC000000390492 and ZINC000043014847 interacted significantly with chosen protein targets of AD in the chosen active sites. The docking score was obtained in the range of − 10.8 to − 6.4 for AChE (4EY7), − 8.7 to − 6.1 in BACE 1 (2HM1), − 10.5 to − 6.3 in MAO-A (2Z5X), and − 8.7 to − 6.2 with NMDA (1PBQ). Among these compounds, against each target the best hit was selected on the basis of docking score and binding energy. The best binding ligands were redocked using glide and the docking scores of the comparable binding poses were determined. Comparison between target protein with potent known drugs/inhibitors in the crystal structures for binding modes and the molecular interactions was done. The binding energies of both hydrophilic as well as hydrophobic interacting residues and their bond length of the best predicted mode with each protein target residue are shown in Table 2. The heatmaps based on binding scores are presented in Fig. 2.

**Table 2:** The structures generated through ChemDraw and binding scores of the best predicted compounds along with their Zinc-ID against each protein target

		Binding score			
Zinc_ID	Structure	4EY7	2HM1	2Z5X	1PBQ
Control		− 11.9	− 9.1	− 7.5	− 8.7
ZINC000043014847		− 10.3	− 8.4	− 8.3	− 8.6
ZINC000002010548		− 10.3	− 8.7	− 10.1	− 8.3
ZINC000000593414		− 10.8	− 7.3	− 8.2	− 8
ZINC000000390492		− 10.1	− 7.9	− 10.5	− 7.9

Where: acetylcholinesterase (4EY7), beta-secretase cleavage enzyme (2HM1), monoamine oxidase (2Z5X) and *N*-methyl-D-aspartate receptor receptor (1PBQ) (Structures are drawn from ChemDraw)

### Validation of docking

**Fig. 3:**
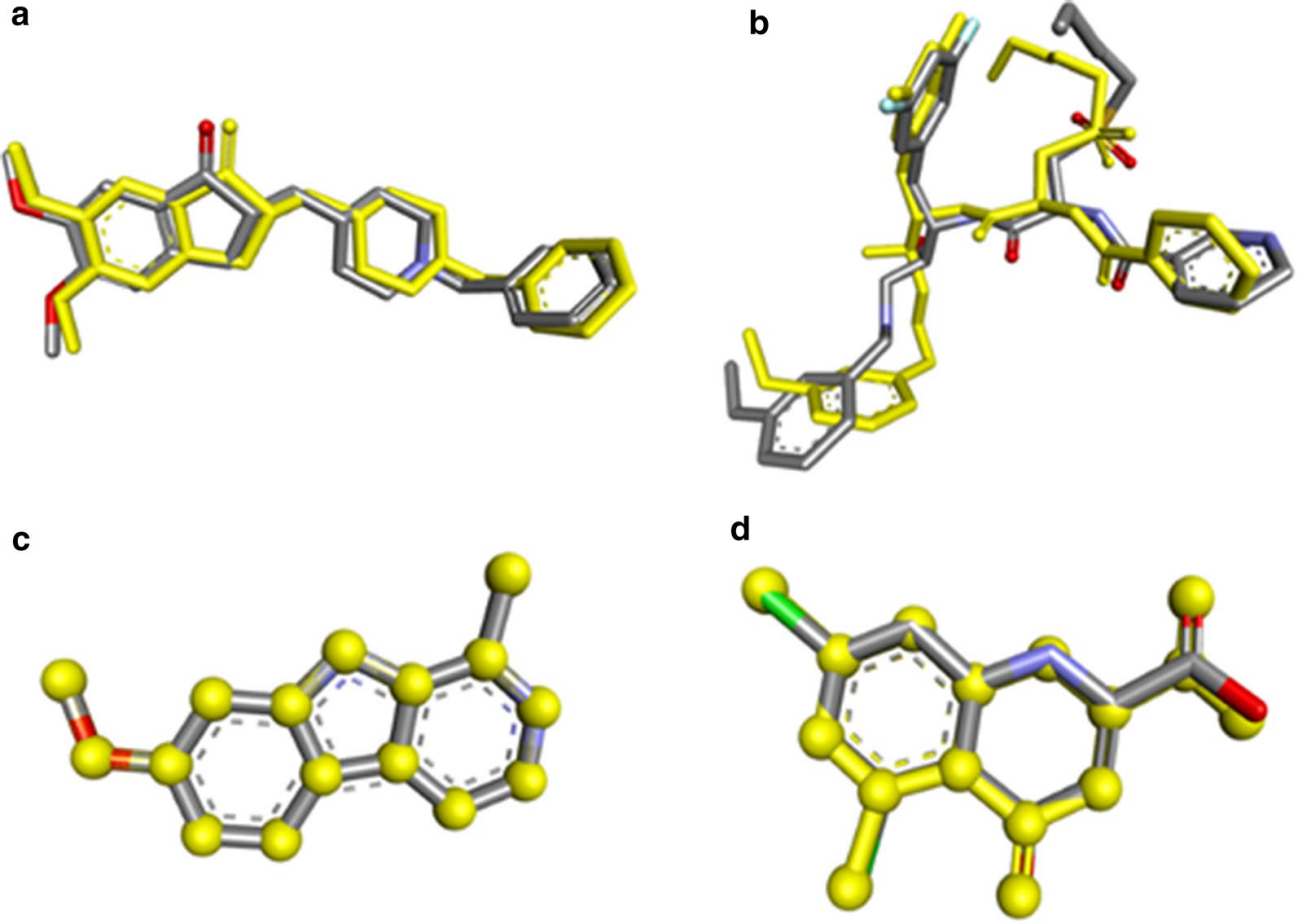
Docking validation by redocking the ligands to their corresponding molecular targets as indicated by their PDB IDs i.e. **a** acetylcholinesterase (4EY7), **b** beta-secretase cleavage enzyme (2HM1), **c** monoamine oxidase (2Z5X) and **d** *N*-methyl-D-aspartate receptor receptor (1PBQ). The original conformation of each ligands is displayed in grey, stick while docked poses are represented in yellow stick

The co-crystallized ligands were extracted from the PDB files of target proteins. Theses extracted ligands were re-docked into the proteins by using same parameters and workflow to validate the reliability and reproducibility of the docking results. The RMSD values of the docked ligand and the co-crystallized ligand was calculated by using all atoms in discovery studio visualizer v17.2. The docked ligand and the co-crystallized structures almost superimpose (Fig. 3) each other and RMSD values ranges from 0.0184 to 0.0992 Å. These results indicate that the docking experiment has produced correct docking poses thus validating the results.

### Molecular interaction of ZINC000000593414 with acetylcholinesterase (4EY7)

**Fig. 4:**
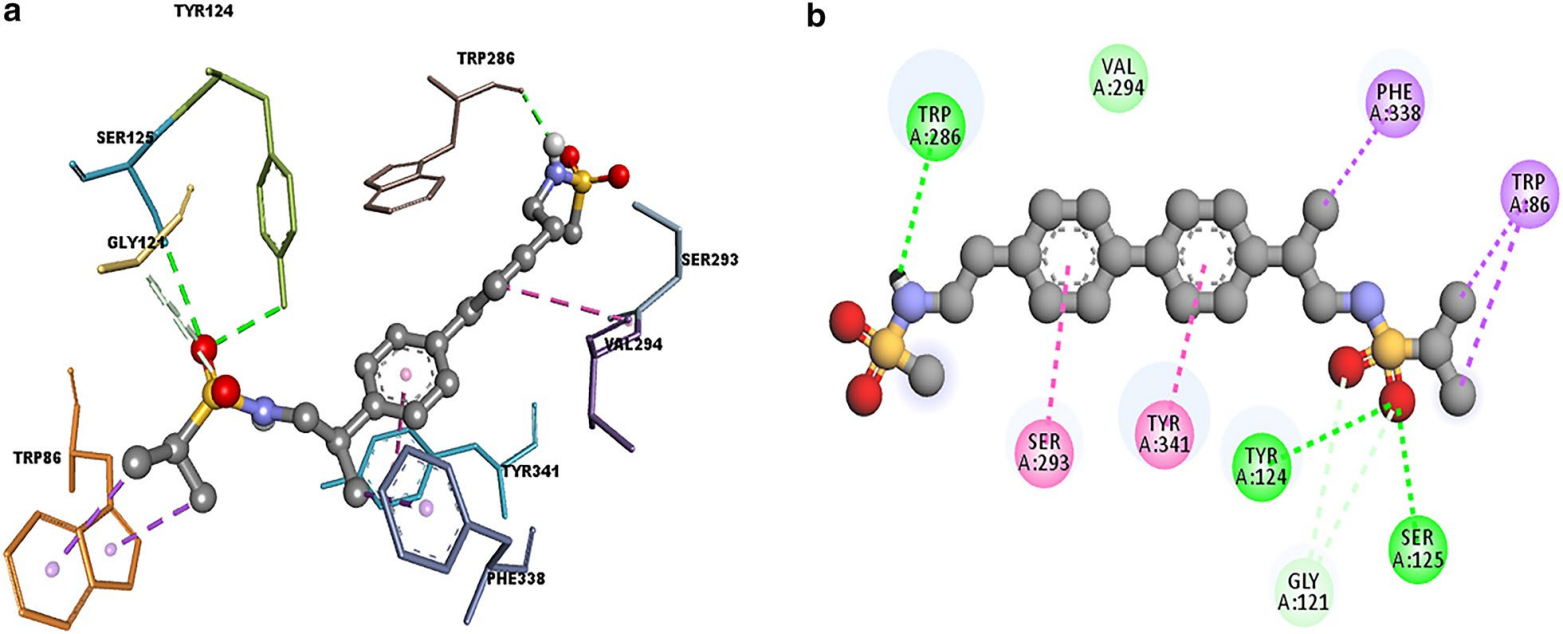
Docking analysis of ZINC000000593414 with acetylcholinesterase (4EY7) depicting the ligand and protein interaction at the active site. The secondary structure of the protein is shown as a solid grey ribbon. Multicolor dots and lines represent key residues. In each fig **a** represents Two dimensional (2D) interaction between ligand and macromolecule and the legend represents the interaction type between the amino acid of the macromolecule and the ligand atoms. The **b** shows the three-dimensional (3D) binding of drug with macromolecule

Against AChE, the lowest binding energy was observed with ZINC000000593414 with binding energy of—10.8 which is comparable with known inhibitor donepezil whosebinding energy was − 11.9. ZINC000000593414 forms hydrogen bonding with Tyr124, Ser125 and Trp286 (PAS residue); pi–pi stacking with Ser293 and Trp341 (PAS residue). Pi-sigma interactions were observed with Trp86 (quaternary ammonium binding locus) and Phe338 (Fig. 4). When redocked with glide, the binding score of the best binding pose was found to be − 8.547.

### Molecular interaction of ZINC000002010548 with β-secretase cleavage enzyme (2HM1)

**Fig. 5:**
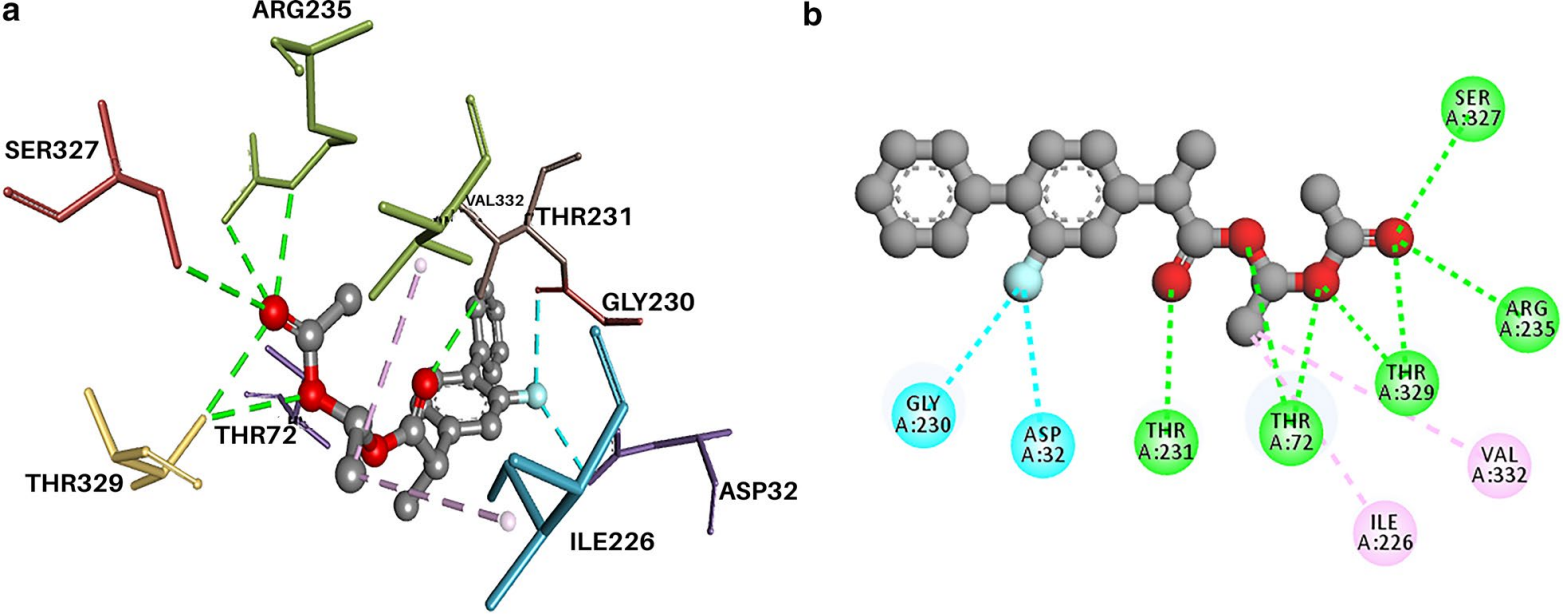
Docking analysis of benperidol and anisoperidone with beta-secretase cleavage enzyme (2HM1) depicting the ligand and protein interaction at the active site. The secondary structure of the protein is shown as a solid grey ribbon. Multicolor dots and lines represent key residues. In each fig **a** represents Two dimensional (2D) interaction between ligand and macromolecule and the legend represents the interaction type between the amino acid of the macromolecule and the ligand atoms. The **b** shows the three-dimensional (3D) binding of drug with macromolecule

The BACE-1 ligand interaction largely depends on the conformation of the active site residues, which consisted of the catalytic dyad (Asp32 and Asp 228), composition of the 10 s loop consist of residues from 9 to 14, flap consisting of 67–77 amino acid and all other residues within 8 Å from aspartates. With BACE-1, ZINC000002010548 exhibited lowest binding energy of—8.7. Hydrogen bonding was formed with Thr329, Thr72, Thr231, Arg235 and Ser327. Salt bridge with Asp32 (catalytic residue) was observed at Phe108 (Fig. 5). When redocked using glide, the docking score was − 5.443.

### Molecular interaction of ZINC000000390492 with monoamine oxidase (2Z5X)

**Fig. 6:**
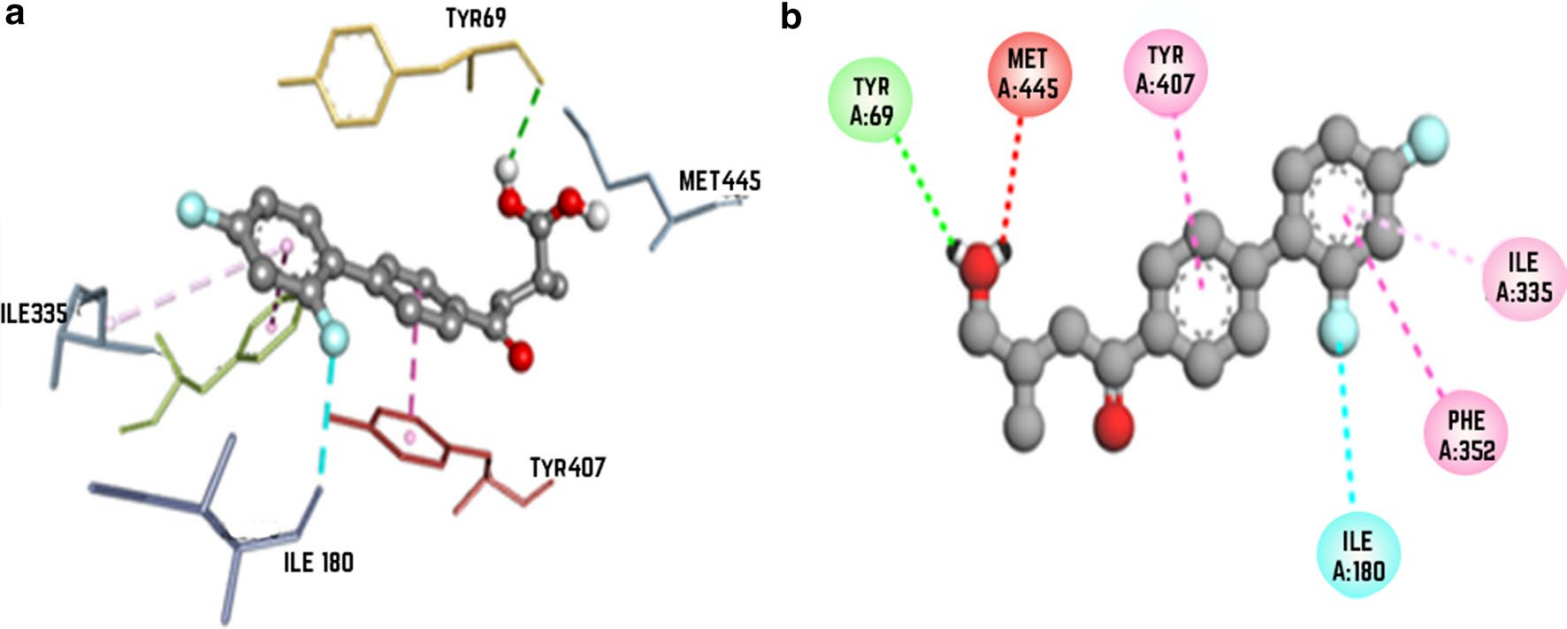
Docking analysis of melperone with monoamine oxidase (2Z5X) depicting the ligand and protein interaction at the active site. The secondary structure of the protein is shown as a solid grey ribbon. Multicolor dots and lines represent key residues. In each fig **a** represents Two dimensional (2D) interaction between ligand and macromolecule and the legend represents the interaction type between the amino acid of the macromolecule and the ligand atoms. The **b** shows the three-dimensional (3D) binding of drug with macromolecule

The results of MAO-A docking showed the least docking score at − 10.5 with ZINC000000390492. Docking results showed hydrogen bonding with Tyr69, Pi–Pi stack with Phe352 and Tyr407 and Pi-alkyl interactions with Ile335 (Fig. 6). When redocked by using glide, the docking score was found to be − 10.498.

### Molecular interaction of ZINC000043014847 with *N*-methyl-D-aspartate receptor receptor (1PBQ)

**Fig. 7:**
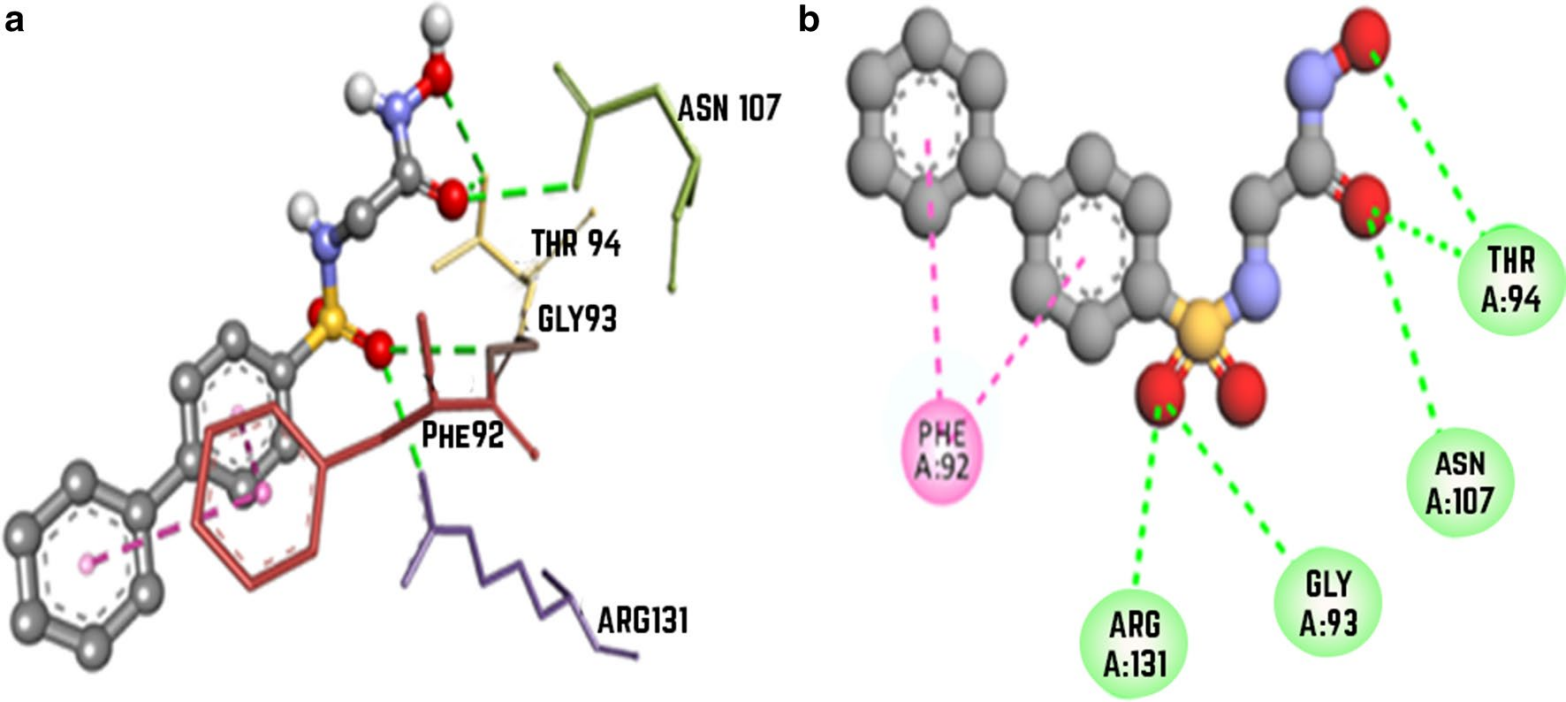
Docking analysis of anisopirol with *N*-methyl-D-aspartate receptor receptor (1PBQ) depicting the ligand and protein interaction at the active site. The secondary structure of the protein is shown as a solid grey ribbon. Multicolor dots and lines represent key residues. In each fig **a** represents Two dimensional (2D) interaction between ligand and macromolecule and the legend represents the interaction type between the amino acid of the macromolecule and the ligand atoms. The **b** shows the three-dimensional (3D) binding of drug with macromolecule

ZINC000043014847 showed the best docking score of—8.6 whereas DCKA showed a docking score of − 8.7. Hydrogen bonding was observed between the ligand and Gly93, Thr94, Asn107, Arg131 whereas pi–pi stacking was observed with Phe92 of 1PBQ (Fig. 7). When redocked by using glide, the docking score was found to be − 6.233.

### Prediction of pharmacokinetic properties

**Fig. 8:**
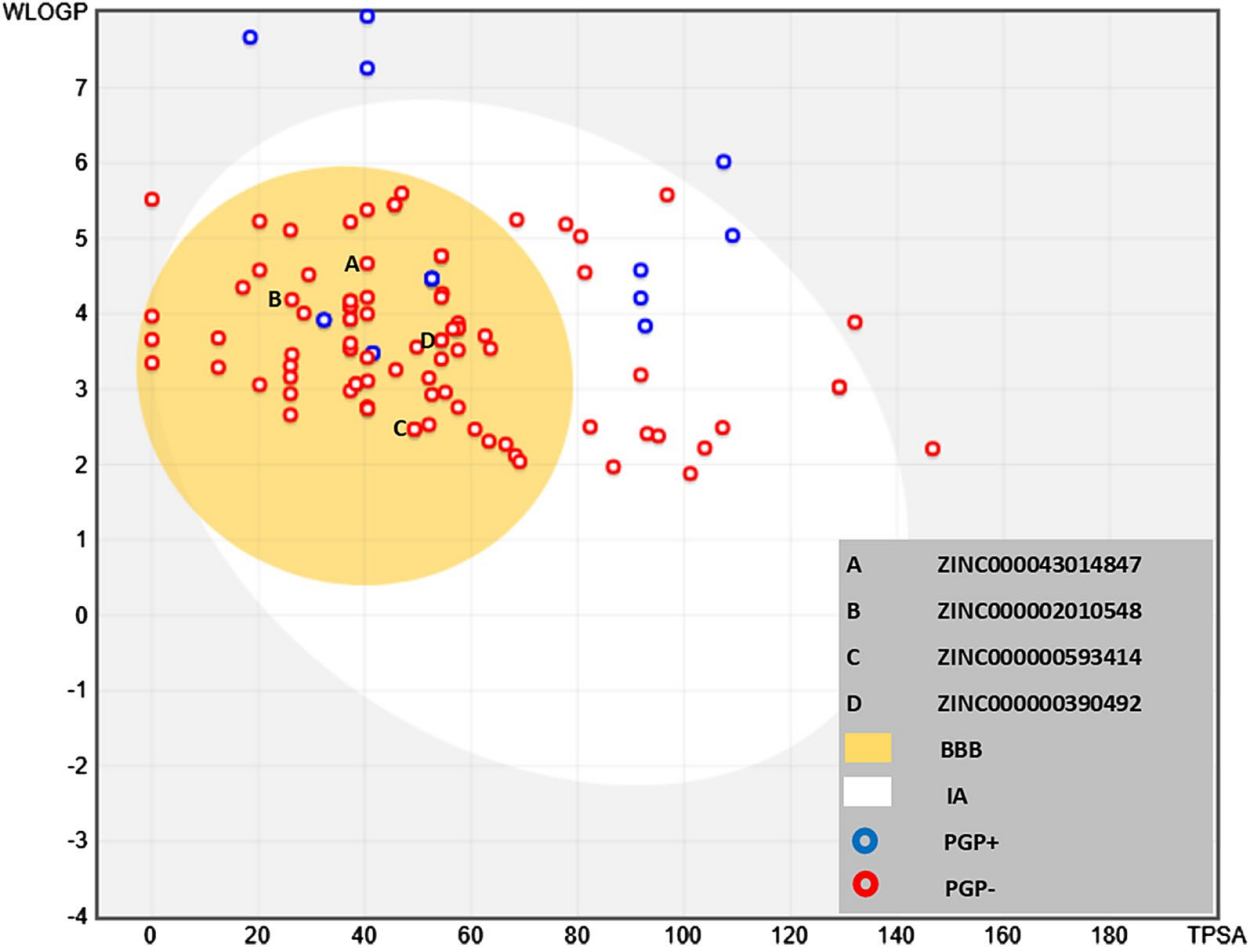
The boiled egg model to graphically represent the intestinal absorption and the brain penetration of the ligands as function of lipophilicity and polar surface area of the molecules. The molecules (represented as dots) within the yellow yolk are well penetrated within the brain with good intestinal absorption. The molecules represented with blue dots could be a substrate for P-Glycoprotein, reducing their absorption and penetration within brain

In regards to prediction of pharmacokinetic properties, none of the compounds in current study demonstrate violation of the Lipinski’s rule of five. The percentage of oral absorption of drug in human was calculated on the scale of 0–100% to predict the oral absorption of the drug. Absorption of more than 80% was considered as good absorption whereas any compound having less than 25% absorption is poor. According to this principle; all the drugs when given via oral route have medium to high absorption. Brain availability by crossing the blood brain barrier was also found to be from medium to high as represented by boiled egg model (Fig. 8). This model gives a nice and simple graphical representation of intestinal absorption and brain penetration of the ligands as a function of lipophilic nature (WLOGP) and polarity of the molecules (TPSA).

## Discussion

Molecular docking is an important technique. It is actually not a standalone technique but works best if treated as a supplementary technique along with other in silico methods as well as in vitro and in vivo experiments [28]. While there is a dire need for new pharmaceutical research in this field, in silico drug analysis is an effective and promising tool for discovering therapeutic utility of both new and already existing drugs [29]. There are number of examples of repurposed drugs which were discovered by the in silico approach and are now being used in many diseases [23] including AD [30].

The main focus of the present study is to signify importance of docking analysis and identify compounds containing biaryl scaffold for the management of AD. About, 107 compounds were screened using docking. Among screened compounds significantly interacting compounds with selected protein targets of AD were identified.

Some previous studies showed the possible potential of marketed antipsychotic drugs against various targets associated with AD by using docking approach [23]. Previously conventional structure-based docking method for the identification of drug molecules for BACE were largely futile [31]. By using in silico computer-aided drug design approaches, the activity of biaryl compounds against AD targets BACE1 and BACE2 was also determined and interestingly it was found that the fused-ring compounds are in general more active than the biaryl-based ligands [32].

In present study 4 targets of AD were chosen. The results of docking were firstly validated. As a general rule, the success of the docking scoring function is validated if the conformation of the bound ligand in the crystal structure resembles the conformation of the docked ligand [33].

One of the selected targets was AChe. An enzyme Ache catalyzes the metabolism of acetylcholine and some other choline esters neurotransmitters. Number of well-known drugs interact with acetylcholinesterase [34]. The human AChE’s active site is 20 Å deep. The active site comprises of catalytic site of AChE (Glu334,Ser 203 and His447), acyl-binding pocket (Phe297and Phe295), oxyanion hole (Ala204, Gly120 and Gly121), quaternary ammonium binding locus (Trp86) and finally, PAS (Tyr341, Trp286, Tyr124, Tyr72 and Asp74), which groups at the active site gorge’s entry [35].

Similarly, monoamine oxidase (MAO) an enzyme majorly involves in the oxidation of numerous vital monoamine hormones and neurotransmitters such as adrenaline, noradrenaline, dopamine, and serotonin [36]. There is a hydrophobic cavity in MAO-A which has a volume of ~ 400 Å. The structure of MAO-A consist of one larger cavity or a bipartite cavity depending upon the conformation of Phe208, but in this case it fails to work as gating residue. The MAO-A has conserved active site residues which comprise of a pair of Tyr of the “aromatic sandwich” and a Lys-hydrogen bonded to the N(5) position of the Flavin i.e. Lys305 in enzyme [37]. There are additional non-conserved active site residues primarily Ile180 and Asn181in MAO-A. The Phe208–Ile335 in MAO-A is the main factor in controlling the differential inhibitor and substrate specificities of these enzymes [38]. In MAO-A, the least docking score was − 10.5 with ZINC000000390492 and in the case of known inhibitor, Hermine, the docking score was − 7.5.

The inhibition of BACE1 through the development of selective and potent inhibitors has been in a limelight in the quest for treatment of AD. BACE an enzyme playing vital role in the proteolytic cleavage of APP is an important target of AD management [39]. The BACE-1 ligand ZINC000002010548 exhibited highest docking score of—8.7 and known inhibitor LY2886721 had docking score of − 9.1 in the previous study [40]

Another target, for protein 1PBQ an ion channel protein and glutamate receptor found in nerve cells, has vital interacting residues with 5,7-Dichlorokynurenic acid (DCKA); a selective NMDA antagonist. DCKA: The co-crystallized ligand interacts with Pro124, Ser180, Arg131 and Thr126. 1PBQ’s hydrophobic pocket has following amino acid residues: Phe92, Phe16, Phe250 and Trp223 [41].

Pharmacokinetic properties of drugs is a significant parameter in drug selection and to determine its utility as a clinically beneficial agent. Docking models have been simulated as a useful alternative to wet lab research procedures especially at initial stages were chemical structures are numerous but resources are scarce [42]. Hence, in present study the physio-chemical factors were determine to assess the ADME properties of the drugs. Lipinski’s rule of five required that the drug must have molecular weight of 500 Da or less, donorHB ≤ 5, accptHB ≤ 10 and octanol–water partition coefficient log P < 5. If drug follow these rules then it’s considered as an orally active drug. The molecules which fails to follow more than one of above stated rules may face difficulty with bioavailability. In current study none of the compounds is violating Lipinski rule of 5. Therefore, in silico computational analysis has been able to identify some encouraging compounds which may prove to be useful in the management of AD. Further research is needed in this regard.

## Conclusion

AD is a complex disease involving many different pathways and drug targets, for instance, AChE, BACE-1, MAO-A and NMDA. In addition to these targets, anti-inflammatory and antioxidant drugs are also proved to be beneficial. Several molecules belonging to different chemical classes have already been developed against these individual targets to relieve the symptoms of this ailment but a multi-target approach is required. In this context the present study explored the potential of biaryl scaffold to inhibit these multiple targets. Of all the compounds screened, biaryl sulphonamides were found to be the top candidate for the cholinergic (AChE), beta-secretase cleavage enzyme (BACE-1), monoaminergic (MAO-A) and glutamatergic system (NMDA). Further analogues can also be computationally designed and tested against these druggable targets. Hence, virtual screening can successfully identify auspicious compounds which might be worthwhile therapeutically in AD.

## Additional file

**Additional file 1.** The ids of compounds searched from zinc data base along with their binding affinities.

## Abbreviations

ADME: absorption, distribution, metabolism, and excretion
AcceltHB: number of hydrogen bond acceptors
4EY7: acetylcholinesterase
AChE: acetylcholinesterase
Ala: alanine
AD: Alzheimer’s disease
Aβ: amyloid beta
APP: amyloid precursor protein
Arg: arginine
Asn: asparagine
Asp: aspartic acid
Aβ40: Aβ of 40 amino acid residues
BACE1: beta-secretase-1
2HM1: beta-secretase cleavage enzyme
CSF: cerebrospinal fluid
CADD: computer-aided drug design
Cys: cysteine
DCKA: 5,7-Dichlorokynurenic acid
DonorHB: number of hydrogen bond donors
Glu: glutamic acid
Gln: glutamine
Gly: glycine
His: histidine
Ile: isoleucine
Leu: leucin
Lys: lysine
Met: methionine
2Z5X: monoamine oxidase
1PBQ: *N*-methyl-D-aspartate receptor
MAO: monoamine oxidases
MW: molecular weight
NFT: neurofibrillary tangles
NMDA: *N*-methyl-D-aspartate receptor
Phe: phenylalanine
Pro: proline
PDB: Protein Data Bank
QPlogPo/w: predicted octanol/water partition coefficient
ROS: reactive oxygen species
Ser: serine
Thr: threonine
Trp: tryptophan
Tyr: tyrosine
Val: valine

## References

[1] Brookmeyer R, Johnson E, Ziegler-Graham K, Arrighi HM (2007). Forecasting the global burden of Alzheimer’s disease. Alzheimers Dement..

[2] Murphy MP, LeVine H (2010). Alzheimer’s disease and the amyloid-β peptide. J Alzheimers Dis.

[3] Kumar S, Chowdhury S, Kumar S (2017). In silico repurposing of antipsychotic drugs for Alzheimer’s disease. BMC Neurosci..

[4] Basile Livia (2017). Virtual Screening in the Search of New and Potent Anti-Alzheimer Agents. Neuromethods.

[5] Kalaria RN, Galloway PG, Perry G (1991). Widespread serum amyloid P immunoreactivity in cortical amyloid deposits and the neurofibrillary pathology of Alzheimer’s disease and other degenerative disorders. Neuropathol Appl Neurobiol.

[6] Janelidze S, Zetterberg H, Mattsson N, Palmqvist S, Vanderstichele H, Lindberg O (2016). CSF Ab42/Ab40 and Ab42/Ab38 ratios: better diagnostic markers of Alzheimer disease. Ann Clin Transl Neurol..

[7] Gu L, Guo Z (2013). Alzheimer’s Aβ42 and Aβ40 peptides form interlaced amyloid fibrils. J Neurochem.

[8] Coyle JT, Price DL, DeLong MR (1983). Alzheimer’s disease: a disorder of cortical cholinergic innervation. Am Assoc Adv Sci..

[9] García-Ayllón M-S (2011). Revisiting the role of acetylcholinesterase in Alzheimer’s disease: cross-talk with P-tau and β-amyloid. Front Mol Neurosci..

[10] Huang WJ, Zhang X, Chen WW (2016). Role of oxidative stress in Alzheimer’s disease. Biomed Res Int.

[11] Son SY, Tsukihara T, Ma J, Kondou Y, Yoshimura M, Yamashita E (2008). Structure of human monoamine oxidase A at 2.2-Å resolution: the control of opening the entry for substrates/inhibitors. Proc Natl Acad.

[12] Butterfield DA, Pocernich CB (2003). The glutamatergic system and Alzheimer’s disease: therapeutic implications. CNS Drugs.

[13] Parsons CG, Danysz W, Dekundy A, Pulte I (2013). Memantine and cholinesterase inhibitors: complementary mechanisms in the treatment of Alzheimer’s disease. Neurotox Res.

[14] Zhang Y, Li P, Feng J, Wu M (2016). Dysfunction of NMDA receptors in Alzheimer’s disease. Neurol Sci..

[15] Goodsell DS, Morris GM, Olson AJ (1996). Automated docking of flexible ligands: applications of AutoDock. J Mol Recognit.

[16] Baig MH, Ahmad K, Roy S, Ashraf JM, Adil M, Siddiqui MH (2016). Computer aided drug design: success and limitations. Curr Pharm Des.

[17] Cheung J, Rudolph MJ, Burshteyn F, Cassidy MS, Gary EN, Love J (2012). Structures of human acetylcholinesterase in complex with pharmacologically important ligands. J Med Chem.

[18] Mirsafian H, Mat Ripen A, Merican AF, Bin Mohamad S (2014). Amino acid sequence and structural comparison of BACE1 and BACE2 using evolutionary trace method. Sci World J.

[19] De Colibus L, Li M, Binda C, Lustig A, Edmondson DE, Mattevi A (2005). Three-dimensional structure of human monoamine oxidase A (MAO A): relation to the structures of rat MAO A and human MAO B. Proc Natl Acad Sci U S A..

[20] Hedegaard M, Hansen KB, Andersen KT, Bräuner-Osborne H, Traynelis SF (2012). Molecular pharmacology of human NMDA receptors. Neurochem Int.

[21] Pettersen EF, Goddard TD, Huang CC, Couch GS, Greenblatt DM, Meng EC (2004). UCSF Chimera–a visualization system for exploratory research and analysis. J Comput Chem.

[22] Shelley JC, Cholleti A, Frye LL, Greenwood JR, Timlin MR, Uchimaya M (2007). Epik: a software program for pK(a) prediction and protonation state generation for drug-like molecules. J Comput Aided Mol Des.

[23] Kumar A, Bora U (2012). In silico inhibition studies of NF-κB p50 subunit by curcumin and its natural derivatives. Med Chem Res.

[24] Dallakyan S, Olson AJ (2015). Small-molecule library screening by docking with PyRx. Methods Mol Biol Clifton NJ..

[25] Sander T, Freyss J, von Korff M, Rufener C (2015). DataWarrior: an open-source program for chemistry aware data visualization and analysis. J Chem Inf Model.

[26] Backman TWH, Cao Y, Girke T (2011). ChemMine tools: an online service for analyzing and clustering small molecules. Nucleic Acids Res.

[27] Daina A, Michielin O, Zoete V (2017). SwissADME: a free web tool to evaluate pharmacokinetics, drug-likeness and medicinal chemistry friendliness of small molecules. Sci Rep..

[28] Goodsell David S, Morris Garrett M, Olson Arthur J (1996). Automated docking of flexible ligands: applications of AutoDock. J Mol Recognit.

[29] Merk D, Grisoni F, Friedrich L, Gelzinyte E, Schneider G (2018). Scaffold hopping from synthetic RXR modulators by virtual screening and *de novo* design. MedChemComm..

[30] Roy S, Kumar A, Baig MH, Masařík M, Provazník I (2015). Virtual screening, ADMET profiling, molecular docking and dynamics approaches to search for potent selective natural molecules based inhibitors against metallothionein-III to study Alzheimer’s disease. Methods San Diego Calif..

[31] Manoharan P, Ghoshal N (2018). Fragment-based virtual screening approach and molecular dynamics simulation studies for identification of BACE1 inhibitor leads. J Biomol Struct Dyn.

[32] Chirapu SR, Pachaiyappan B, Nural HF, Cheng X, Yuan H, Lankin DC (2009). Molecular modeling, synthesis, and activity studies of novel biaryl and fused-ring BACE1 inhibitors. Bioorg Med Chem Lett.

[33] Hevener KE, Zhao W, Ball DM, Babaoglu K, Qi J, White SW (2009). Validation of molecular docking programs for virtual screening against dihydropteroate synthase. J Chem Inf Model.

[34] Baig MH, Rizvi SMD, Shakil S, Kamal MA, Khan S (2014). A neuroinformatics study describing molecular interaction of Cisplatin with acetylcholinesterase: a plausible cause for anticancer drug induced neurotoxicity. CNS Neurol Disord Drug Targets.

[35] Johnson G, Moore SW (2006). The peripheral anionic site of acetylcholinesterase: structure, functions and potential role in rational drug design. Curr Pharm Des.

[36] Nagatsu T (2004). Progress in monoamine oxidase (MAO) research in relation to genetic engineering. Neurotoxicology..

[37] Geha RM, Chen K, Wouters J, Ooms F, Shih JC (2002). Analysis of conserved active site residues in monoamine oxidase A and B and their three-dimensional molecular modeling. J Biol Chem.

[38] Edmondson DE, Binda C, Wang J, Upadhyay AK, Mattevi A (2009). Molecular and mechanistic properties of the membrane-bound mitochondrial monoamine oxidases. Biochemistry (Mosc)..

[39] John V (2006). Human β-secretase (BACE) and BACE Inhibitors: progress Report. Curr Top Med Chem.

[40] May PC, Willis BA, Lowe SL, Dean RA, Monk SA, Cocke PJ (2015). The potent BACE1 Inhibitor LY2886721 elicits robust central A pharmacodynamic responses in mice, dogs, and humans. J Neurosci.

[41] Furukawa H, Gouaux E (2003). Mechanisms of activation, inhibition and specificity: crystal structures of the NMDA receptor NR1 ligand-binding core. EMBO J.

[42] Daina A, Michielin O, Zoete V (2017). SwissADME: a free web tool to evaluate pharmacokinetics, drug-likeness and medicinal chemistry friendliness of small molecules. Sci Rep..

